# Refinement of Coronary Artery Bypass Grafting at Juntendo University Hospital

**DOI:** 10.14789/jmj.JMJ21-0012-R

**Published:** 2022-02-16

**Authors:** ATSUSHI AMANO

**Affiliations:** 1 Department of Cardiovascular Surgery, Juntendo University, Tokyo, Japan; 1 Department of Cardiovascular Surgery, Juntendo University, Tokyo, Japan

**Keywords:** coronary artery bypass grafting, off-pump coronary artery bypass grafting, atrial fibrillation, stroke, left atrial appendage

## Abstract

Surgical treatment of ischemic heart disease began in 1945. After 1970, coronary artery bypass grafting (CABG) with cardiopulmonary bypass was developed along with coronary angiography. Juntendo University has been treating ischemic heart disease since 1980, and is actively performing off-pump CABG (OPCAB) since 2002. Besides the age of patients undergoing surgery, complications such as chronic hemodialysis, cerebrovascular disease, and malignancies make it challenging to reduce postoperative complications using OPCAB as graft preservation. OPCAB is technically challenging, and the CORONARY trial did not reveal its superiority over conventional CABG. Furthermore, high revascularization rates and technical differences among surgeons are important concerns. While not widely accepted in Europe and the United States, OPCAB comprises 65% of all stand-alone CABG in Japan. Japan reported a 2.5% hospital mortality rate in 2018, while the US reported 2.2% (according to the American Association of Thoracic Surgeons). In contrast, Juntendo University Hospital has maintained a 1% hospital mortality rate since 1984. To reduce the incidence of remote stroke in CABG patients, Juntendo has been using stroke-free management since 2010. Postoperative atrial fibrillation is 4-5 times more likely to recur than normal sinus rhythm after a 5-year course. In our study, 20% of patients suffered from chronic atrial fibrillation after ten years. Furthermore, left atrial appendage closure or amputation significantly reduces stroke in patients who undergo CABG and develop postoperative atrial fibrillation. Thus, OPCAB is a minimally invasive procedure with fewer complications; prevention of cardiogenic cerebral infarction can help improve remote outcomes.

## Introduction

Surgical treatment of ischemic heart disease began in 1945 with the Veinberg operation, in which the left internal thoracic artery (LITA) was implanted into the myocardium^1)^, followed by direct coronary revascularization with the great saphenous vein (SVG) in 1962^2)^. In 1969, Favaloro et al. reported the surgical results of 100 coronary artery bypass grafting (CABG) cases. The surgical mortality was excellent at 5%; LITA was used in 40% of cases, and graft patency was favorable in 80% of cases in one year^3)^. After 1970, CABG under the arrested heart with cardiopulmonary bypass was the conventional procedure performed. Rapid advancements occurred in CABG and coronary angiography (CAG), which were developed in the same era. In 1975, the advantages of CABG over medical therapy were confirmed, particularly for left main trunk disease^4)^. Since 1980, Juntendo University Hospital has been treating ischemic heart disease. Since 2002, we introduced off-pump CABG (OPCAB) technique and have been the world leader of the filed. In this review, we described our surgical strategy for ischemic heart disease along with the world trend of the field.

## The Continuing Evolution of Bypass Grafts

**Figure 1:**
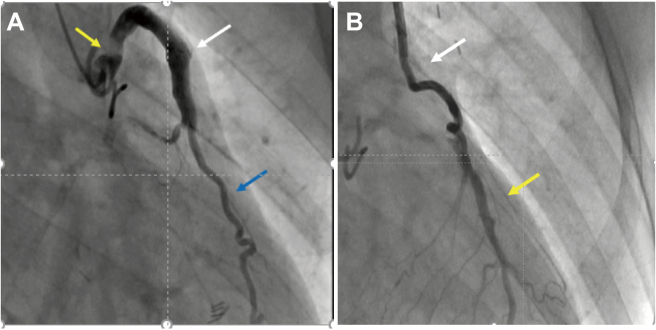
Postoperative cineangiography 1A Saphenous vein graft to left circumflex artery White allow is Saphenous vein graft. Blue allow is left circumflex artery Yellow allow is SVG disease due to neointima and atherosclerosis. 1B Left internal thoracic artery to left anterior descending artery White allow is left internal thoracic artery。Yellow allow is l left anterior descending artery

**Figure 2:**
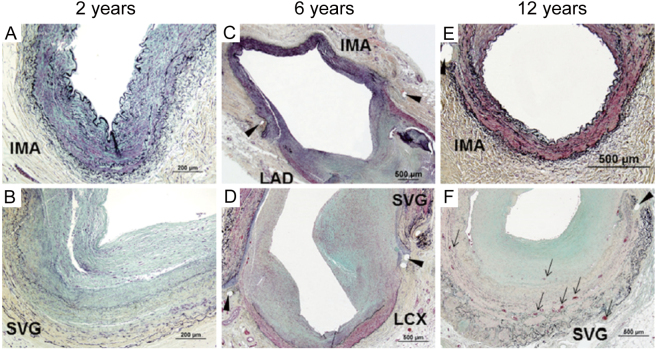
Changes of microscopic findings of IMA and SVG Internal mammary artery (IMA) saphenous vein graft (SVG). A,B. Histologic sections showing IMA and SVG obtained from a 76-year-old man who underwent coronary artery bypass graft (CABG) surgery 2 years. A: IMA shows no or rare intimal smooth muscle cells (SMCs) B: SVG exhibit moderate neointimal growth with few SMCs but rich in matrix which consists of proteoglycans and collagen. C,D: Histologic sections showing IMA and SVG obtained from a 69-year-old man who underwent CABG surgery 6 years ago. No intimal thickening in IMA C: The presence of moderate neointimal thickening in SVG from SMCs and proteoglycan-collagenous matrix at the site of anastomosis (arrow heads indicate suture sites) with left anterior descending artery (LAD) or left circumflex artery (LCX) E,F: IMA graft and SVG from a 77-year-old woman who underwent CABG surgery 12 years ago . While the IMA shows minimal intimal thickening, SVG exhibits moderate to severe neointimal growth with proteoglycan-collagen matrix and angiogenesis (arrows). Reprinted from Otsuka F, Yahagi K, Sakakura K. Why is the mammary artery so special and what protects it from atherosclerosis? Ann Cardiothorac Surg, 2013; 2: 519-526.

A series of reports in the 1980s and 1990s showed that regarding long-term outcomes, LITA graft was superior to SVG graft^5, 6)^, due to the superior endothelial function of LITA, which had beneficial physiological and metabolic effects not only on the graft itself but also on the anastomosed coronary artery^7)^. By reviewing the CAG (Figures 1A and 1B) and pathological findings (Figure 2) of LITA and SVG after more than a decade, it has been found that intimal thickening of the SVG is an integral part of the problem^8)^. The utility of multiple arterial grafts has been reported^9)^, and the right gastric epigastric artery graft devised by Suma et al. is excellent for revascularizing the right coronary artery^10)^. It also has good long-term patency with an 87% 5-year patency rate^11, 12)^. The radial artery was also used as a graft, and good results were demonstrated with an innovative harvesting method^13)^, and an excellent 10-year patency rate of 83%^14)^. However, there is still disagreement whether the right internal thoracic artery (RITA) or radial artery is better as a second graft^15)^.

In our department, the 10-year patency rate of LITA graft to the left anterior descending branch was 96%, and the 10-year patency rates of RITA and SVG grafts to the left circumflex artery region were 86% and 82%, respectively. In our laboratory, Yokoyama et al. conducted a study regarding spasm of gastroepiploic artery graft (GEA) and confirmed that denervation by periarterial tissue resection prevented the arterial spasm of the graft^16)^. In addition, we selected grafts based on the atherosclerotic status of the right coronary artery. In our institution, the 10-year patency rates of GEA and SVG were 86% and 84%, respectively. Based on our previous analysis, we predicted that the mean survival time of SVG was 13-14 years.

Between 1990 and 2000, there was an increase in the number of reports on the benefits of using RITA and LITA, and meta-analyses confirmed the evidence for their efficacy^6, 17, 18)^. Conversely, most surgeons struggled with multidrug-resistant *Staphylococcus aureus* (MRSA) infection, and the complications of mediastinitis when using bilateral internal thoracic arteries were challenging to treat and could be fatal. In addition, complications associated with MRSA infection increased both the cost and length of hospitalization. In response, a new method of harvesting arterial grafts has been evolving, and its anatomical characteristics and preventive effect on mediastinitis have been reported^19, 20)^. This harvesting technique is called skeletonized harvesting, which removes most of the tissue surrounding the LITA. This technique extends the length of the harvested graft by 2-3 cm and increases the number of anastomotic sites, allowing for various graft designs^21)^.

## Changes in Lifestyle and Coronary Artery Reconstruction in the Japanese Population

Since 2000, the prevalence of lifestyle-related diseases (e.g., diabetes and hyperlipidemia) and metabolic syndromes in Japan has risen to levels comparable to those in Western countries. When diabetes and hyperlipidemia are poorly controlled, the coronary arteries may show extensive calcification and unstable plaque or complicated by multiple lesions. Although the results of coronary stenting are stable, the frequency of restenosis is comparatively high in these patients. Since 2005, drug-eluting stents with low restenosis rates and potent antiplatelet agents have been commonly used to improve long-term outcomes^22)^. Recently, we have used the Syntax score, which focusses on the complexity of the coronary lesion. The results showed that percutaneous coronary intervention was more effective when the Syntax score was low. However, CABG is still superior for intermediate and high Syntax scores^23)^; the efficacy of CABG for treating narrow coronary arteries and complete chronic occlusions due to diffuse atherosclerosis was remarkable, particularly in patients with diabetes and poor lipid management^23)^. In recent years, the ability to analyze images has become more sophisticated, and we can now use intravascular ultrasound, fractional flow reserve analysis, and cardiac nuclear medicine to select the most appropriate treatment and graft for patients^24)^. Although there are no long-term results published yet, we believe that the usefulness of these methods will become apparent in the future.

Since 2010, the problem in Japan has been the aging of patients undergoing surgery and the increase in complex systemic conditions such as chronic dialysis, cerebrovascular disease, and malignant complications. Therefore, we need to reduce postoperative complications, life expectancy, and graft patency rates.

## Approaches to the Treatment of Coronary Artery Disease at Juntendo University

**Figure 3:**
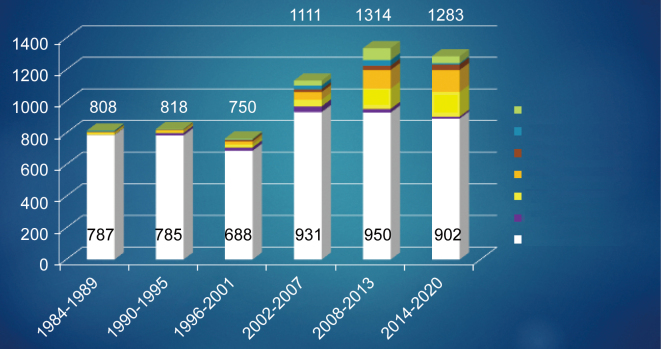
Changes in the Number of Surgeries for Ischemic Heart Disease at Juntendo University CABG: coronary artery bypass grafting AVR: aortic valve replacement LV plasty: left ventricular plasty

**Figure 4:**
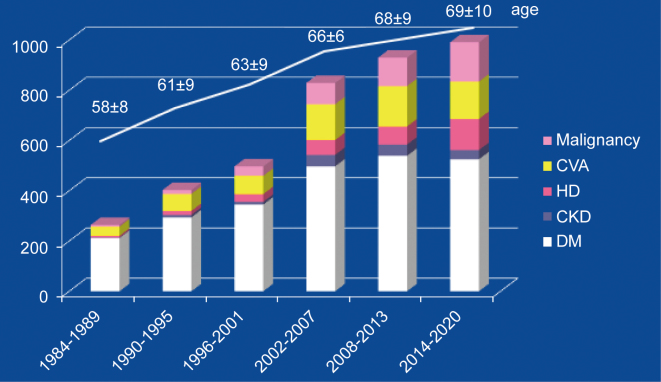
Primary isolated coronary artery bypass grafting at Juntendo University Changes of preoperative patients` baseline CVA: cerebrovascular accident, HD; hemodialysis, CKD: chronic kidney disease, DM: Diabetes Mellitus

Since 1980, coronary artery bypass surgery has been actively performed for ischemic heart disease at Juntendo University. Since the establishment of the Department of Cardiovascular Surgery in 2002, the number of coronary artery surgery cases has increased, and in tandem, the number of associated complications. In particular, the number of operations for aortic valve disease is increasing (Figure 3). From 2000 to 2020, the average age of patients who underwent primary isolated CABG had increased from 66 years to 69 years. Furthermore, the number of patients on dialysis and those with cerebral or malignant disease had increased, accounting for 14%, 16%, and 17% of all patients, respectively (Figure 4).

We actively performed off-pump coronary artery bypass grafting (OPCAB) to make CABG less invasive and accommodated the increasing number of such high-risk cases. However, OPCAB is technically difficult, and the CORONARY trial reports in 2012 and 2013 did not reveal its superiority over conventional cardiopulmonary surgery^25, 26)^. A meta- analysis by Deppe et al. (2016) showed that OPCAB was superior in most categories, including mortality, myocardial infarction, stroke, renal failure, infection, and bleeding; however, the large variation of revascularization rate and technical differences among surgeons were major concerns^27)^. Consequently, OPCAB has not become popular in Europe and the United States; however, OPCAB use in Japan comprises up to 65% of all isolated CABG procedures due to its active promotion by leading institutions such as Juntendo University (Table 1).

**Table 1:** Changes in the number of primary isolated coronary artery bypass grafting at Juntendo University

	1984-1989	1990-1995	1996-2001	2002-2007	2008-2012	2013-2020
No. of patients	787	785	688	931	950	902
OPCAB	0	0	32 (4.7%)	912 (97.9%)	950 (97.4%)	902 (98.8%)
MIDCAB	0	0	0	67	15	24
Emergency(<24hrs)	12(1.5%)	16(2.0%)	27(3.9%)	88(9.8%)	53(5.9%)	59(5.9%)
No. of anastomosis	2.4 ± 0.8	2.5 ± 0.8	2.7 ± 0.8	3.6 ± 1.4	3.4 ± 1.3	3.3 ± 1.2
Only SVG	513	84	14	5	8	67
No. of arterial graft						
1	272	676	493	162	164	201
2	0	25	180	232	324	269
3	0	0	1	221	271	219
≥4	0	0	0	311	183	206

No. of patients; Number of patients. OPCAB: Off-pump coronary artery bypass grafting. MIDCAB: Minimally invasive coronary artery bypass grafting. No of anastomoses: Number of anastomoses Only SVG: Only saphenous vein graft. No. of arterial grafts: Number of arterial grafts

**Figure 5:**
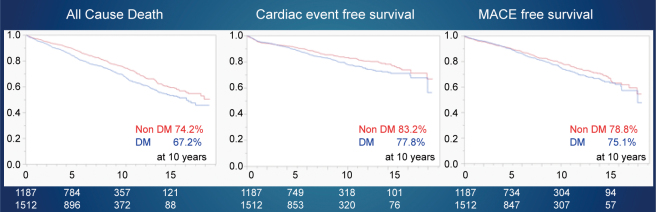
Primary isolated coronary artery bypass grafting at Juntendo University Long-term results after coronary artery bypass grafting DM: patients with diabetes mellitus nonDM: patients without diabetes mellitus

**Figure 6:**
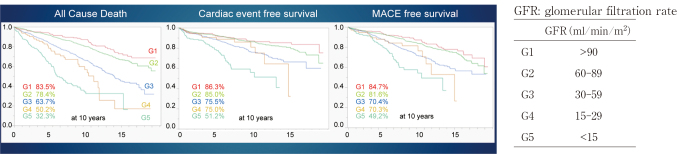
Primary isolated coronary artery bypass grafting at Juntendo University Long-term results in the follow-up of patients with renal dysfunction

Since 2002, we have performed OPCAB in 98% of our patients at Juntendo. The average number of bypasses exceeded three, and multiple arterial grafts were used in over 70% of the cases (Table 1). In 2018, the hospital mortality rates were 2.5% in Japan^28)^, and the American Association of Thoracic Surgeons reported a rate of 2.2%^29)^. In contrast, Juntendo has maintained a 1% in-hospital mortality rate since 1984. Furthermore, neurological complications have reduced from 3-4% in the 1990s to 1%. Since the introduction of OPCAB, there have been fewer infections, higher blood transfusion rates (70%), and shorter hospital stays (Table 2). In the diabetic group, the 10-year overall survival rate was 67%, and the cardiac death-free rate was 78%, which was worse than the non-diabetic groups with rates of 74% and 83%, respectively (Figure 5). Regarding preoperative renal function, all-cause mortality, cardiac mortality avoidance rate, and major adverse cardiovascular events (MACE), outcomes all worsened from CKD stages G1 to G5. The 10-year survival rate for G1 was 84%, but 32% for G5, and long-term outcomes worsened as renal function declined, with 85% for G1 and 49% for G5 MACE (Figure 6). In a meta-analysis of all-cause mortality, the independent risk factors were preoperative dyslipidemia, diabetes mellitus, peripheral vascular disease, previous stroke, dialysis, and low left ventricular function (Table 3). For MACE, the independent risk factors were obesity, dialysis, low left ventricular function, peripheral vascular disease, and stroke function (Table 4).

**Table 2:** Operative results of primary isolated coronary artery bypass grafting at Juntendo University

	1984-1989	1990-1995	1996-2001	2002-2007	2008-2012	2013-2020
No. of patients	787	785	688	931	950	902
POAF	210(26.7%)	232(29.6%)	198(28.8%)	191(20.5%)	309(32.5%)	252(25.7%)
Cerebrovascular accident	21(2.7%)	32(4.1%)	17(2.5%)	17(1.8%)	11(1.2%)	6(0.7%)
Respiratory complications	10(1.3%)	18(2.3%)	12(1.7%)	132(14.2%)	52(5.5%)	57(6.3%)
Renal insufficiency	9(1.1%)	38(4.8%)	54(7.8%)	44(4.7%)	50(5.3%)	65(7.2%)
Infection	31(3.9%)	13(2.0%)	7(1.0%)	19(2.0%)	23(2.4%)	15(1.7%)
No blood transfusion	390(49.6%)	364(46.3%)	356(51.7%)	727(78.1%)	694(73.1%)	619(68.6%)
Hospital stay (days)	28±29	24±14	20＋13	11±7	12±14	13±14
Hospital moratlity	9(1.1%)	16(2.0%)	7(1.0%)	11(1.1%)	7(0.7%)	12(1.1%)

POAF; postoperative atrial fibrillation

**Table 3:** Multivariate predictors for hospital mortality

	Univariate		Multivariate	
	OR (95%CI)	P value	OR (95%CI)	P value
Age>75 (years) (yes=1)	2.17(1.84-2.55)	<0.001	2.03(1.94-2.72)	<0.001
Sex (Male=1)	1.20 (0.97-1.47)	0.09		
Body mass index >30 (yes=1)	0.84(0.52-1.36)	0.48		
Hypertension (yes=1)	1.24(1.03-1.48)	0.02	1.10(0.91-1.33)	0.30
Lipid disorder (yes=1)	0.62(0.53-0.73)	<0.0001	0.67(0.57-0.79)	<0.001
Diabetes Mellitus (yes=1)	1.31(1.12-1.53)	<0.001	1.30(1.11-1.53)	0.001
Peripheral arterial disease (yes=1)	2.00(1.66-2.42)	<0.001	1.69(1.39-2.06)	<0.001
Previous stroke (yes=1)	1.86(1.54-2.24)	<0.001	1.43(1.18-1.73)	<0.001
Chronic kidney disease				
eGFR<60 (ml/min/1.73m^2^) (yes=1)	2.51(2.15-2.94)	<0.001		
Hemodialysis (yes=1)	3.73(2.98-4.65)	<0.001	3.16(2.50-3.99)	<0.001
LVEF<40% (yes=1)	1.81(1.53-2.13)	<0.001	1.69(1.43-2.00)	0.003

LVEF: Left ventricular ejection fraction.

**Table 4:** Multivariate predictors for MACE; Major Adverse Cardiovascular Events

	Univariate		Multivariate	
	OR (95%CI)	P value	OR (95%CI)	P value
Age>75 (years) (yes=1)	1.14(0.91-1.43)	0.24		
Sex (Male=1)	0.92 (0.73-1.16)	0.49		
Body mass index >30 (yes=1)	1.92(1.29-2.85)	0.001	2.03(1.36-3.02)	<0.0001
Hypertension (yes=1)	1.25(1.00-1.56)	0.04	1.13(0.90-1.42)	0.30
Lipid disorder (yes=1)	1.08(0.87-1.33)	0.50		
Diabetes Mellitus (yes=1)	1.11(0.92-1.34)	0.26		
Peripheral arterial disease (yes=1)	1.74(1.36-2.21)	<0.0001	1.55(1.21-1.99)	<0.0001
Previous stroke (yes=1)	1.44(1.14-1.85)	0.003	1.31(1.02-168)	0.03
Chronic kidney disease				
eGFR<60 (ml/min/1.73m^2^) (yes=1)	1.96(1.62-2.36)	<0.001		
Hemodialysis (yes=1)	2.54(1.86-3.45)	<0.001	2.24(1.63-3.07)	<0.001
LVEF<40% (yes=1)	1.46(1.19-1.80)	<0.001	1.38(1.11-1.70)	0.003

LVEF: Left ventricular ejection fraction.

## New Challenges for CABG Strategies

Based on our experience, we believe that cerebrovascular disorders such as stroke after CABG should be prevented. Atrial fibrillation (AF) is a major cause of cerebral infarction, and it is known that 30% of patients develop transient postoperative AF (POAF), even though the preoperative cases of AF are 2%. However, cardiovascular surgeons have not addressed this problem. Therefore, we believe that an immediate strategy for POAF is necessary.

Furthermore, the addition of left atrial appendage (LAA) closure to the OPCAB for preventing cardiogenic stroke in the Emperor's surgery in 2012 was vital. The surgery results were excellent, and His Majesty the Emperor had lived a healthy life for nine years after the surgery without any particular cardiovascular or neurological events. This valuable experience led us to develop a stroke-free management strategy for patients undergoing CABG.

**Figure 7:**
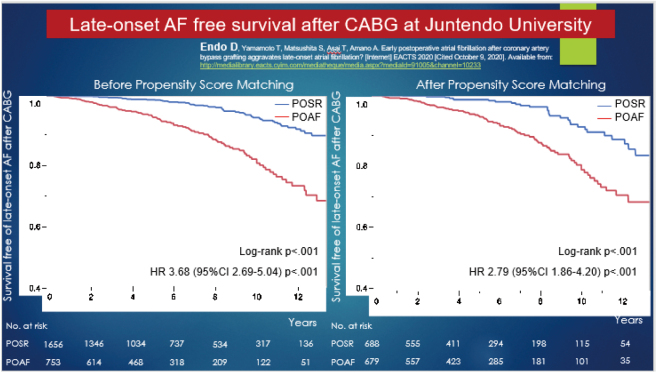
Late-onset AF free survival after CABG at Juntendo University In cases of transient postoperative atrial fibrillation, 20% will develop chronic atrial fibrillation 10 years later.

In the recent years, POAF has been reported as a complication in 30% of CABG, 50% of valvular surgery, 30% of pneumonectomy, and 20% of esophagectomy cases in general surgery^30)^. Moreover, the 2020 European Cardiology Guidelines states that POAF is 4-5 times more likely to recur than normal sinus rhythm after a 5-year follow- up^31)^. These findings have even led to the opinion that pulmonary vein isolation and surgical intervention of the LAA may be desirable in the presence of any risk factor for AF^30)^. Although there is no evidence that POAF tends to convert to distant AF after surgery since the valuable publication in 2014^32)^, Endo and his colleagues found that the recurrence rate of POAF was high and 20% of patients converted to chronic AF at least ten years after surgery (Figure 7)^33)^. Furthermore, the same analysis showed that 8% of all cases older than 70 years had converted to chronic AF.

First performed in 1949, surgical LAA excision is one of the oldest surgical procedures. Since then, many methods have been improved and accomplished, but along with good results, problems have become apparent. Recently, the Society of Thoracic Surgeons 2017 Clinical Practice Guidelines for the Surgical Treatment of Atrial Fibrillation recommended Class IIa for LAA closure^34)^. Specifically, in patients with contraindications to anticoagulation and at a high risk of stroke, left ventricular processing has been determined to be optimal and necessary. A recent study reported that 4,374 (5.8%) of 75,782 patients undergoing cardiac surgery underwent simultaneous left auricular occlusion (LAAO), with a mean follow-up of 2.1 years. They reported that LAAO reduced cerebral infarction and simultaneous LAAO in open-heart surgery reduced all-cause mortality and stroke. However, approximately 75% of the patients had AF. However, this study did not prove the efficacy of LAAO in patients without a history of preoperative AF^35)^.

Advances in preoperative imaging have been remarkable, and preoperative imaging, such as electrocardiogram gated three-dimensional computed tomography and transesophageal echocardiography, is of utmost importance in defining the anatomy of the LAA and is essential for protocol development, including the selection of appropriate devices. Anatomical considerations for preoperative imaging include the size, shape (sharp angles in the form of chicken wings, short neck <10 mm in the form of cauliflower), and presence of comb-like muscles, lobes, and nodes of the LAA. Closure of the LAA can be achieved by catheter-based lumen closure, surgical closure, or resection. Complex morphology can cause difficulties during the insertion of endocardial devices, and the selection of an appropriate method from surgical LAA closure/amputation is desirable. A recent study reported that hemodynamics could predict the risk of thrombus development in the LAA during normal sinus rhythm and AF according to LAA morphology^36)^. The distribution of LAA forms was 30% cactus, 48% chicken wing, 19% windsock, and 3% cauliflower, with a higher incidence of stroke reported in forms other than the chicken wing^37)^. In addition, it has been confirmed that closure of the LAA is more cost-effective than anticoagulation in preventing cerebral infarction in AF^38)^.

## LAA Management for Patients with CABG at Juntendo University

**Figure 8:**
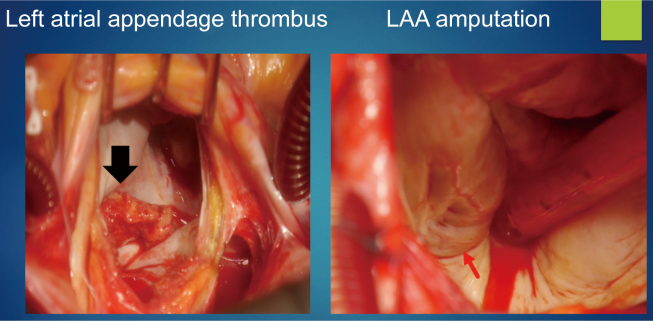
Neointima on the scar site after the left atrial appendage amputation Black arrow is thrombi in Left atrium. Red arrow is neointima. Reprinted from Endo D, Yamamoto T, Kuwaki K et al: Neointima on the scar site after the left atrial appendage amputation. J Card Surg, 2019; 34: 855.

The concept of stroke-free management for CABG surgery at Juntendo University refers to minimizing the invasiveness by OPCAB and preventing AF and cerebral infarction through prophylactic LAA amputation. Since 2012, we have performed LAA closure/amputation in all patients undergoing CABG, even in OPCAB surgery. Endo et al. reported that the intima in the left atrium was smooth, precise, and completely covered after LAA amputation (Figure 8)^39)^. In a survey of 2,268 patients in our department, LAA closure/amputation was associated with no increase in POAF (26%) or bleeding. Furthermore, it effectively prevented cerebral infarction in patients with POAF in the long term and contributed to stroke-free management (Table 5)^40)^.

**Table 5:** Late-onset AF free survival after CABG at Juntendo University

	Multivariate	
	OR (95%CI)	P value
Peripheral arterial disease (yes=1)	2.05(0.92-4.60)	0.08
eGFR<60ml/min.1.73m^2^ (yes=1)	1.30(0.62-2.71)	0.49
Previous stroke (yes=1)	1.54(0.67-3.57)	0.31
LAA amputation(-)/POAF(-)	reference	
LAA amputation(-)/POAF(+)	3.26(1.29-8.26)	0.01
LAA amputation(+)/POAF(-)	1.22(0.43-3.49)	0.72
LAA amputation(+/POAF(+)	0.86(0.18-4.14)	0.85

LAA: Left atrial appendage. POAF: Postoperative atrial fibrillation

**Figure 9:**
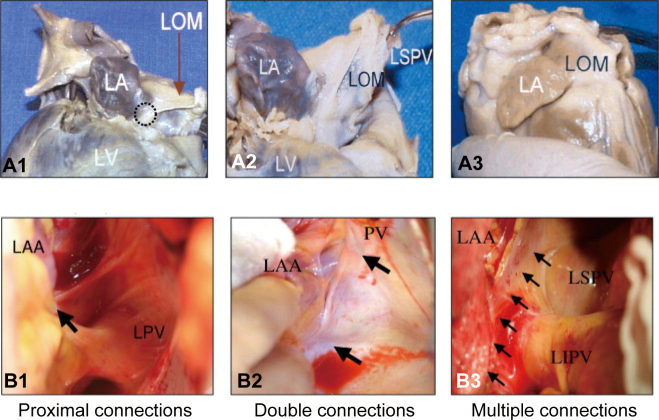
Variations in the ligament of Marshall (LOM) anatomy Variations of LOM(ligament of Marshall) anatomy. A black circle in A1 indicates the proximal connection of the LOM to the CS. A2: A different view of the same heart. The distal end of the LOM inserts into the LSPV. A3: A second heart in which LOM was completely attached to the epicardium. A discrete ligament was not identified. B1: Proximal connection (arrow) between the LOM and the CS. B2: Both the proximal and distal connections of the LOM (2 arrows) in a second heart. B3: A third heart, which seems to have multiple muscle fibers (arrows) connecting the LOM and the LA. Reprinted from Hwang C, Chen PS. Ligament of Marshall: why it is important for atrial fibrillation ablation. Heart Rhythm. 2009; 6(12 Suppl): S35-40.

**Figure 10:**
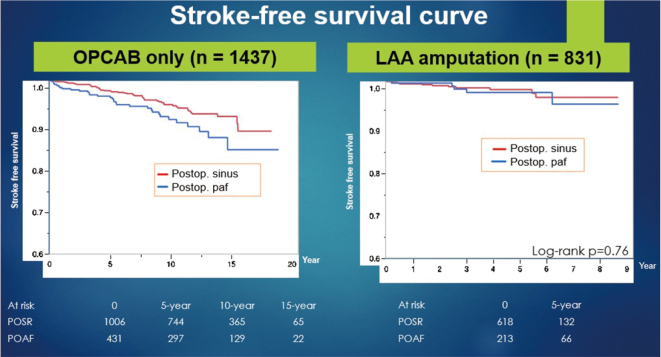
The impact of surgical left atrial appendage amputation/ligation on stroke prevention in patients undergoing off-pump coronary artery bypass grafting. A: Stroke-free survival curve LAA: left atrial appendage POSR: postoperative sinus rhythm POAF: postoperative atrial fibrillation

**Figure 11:**
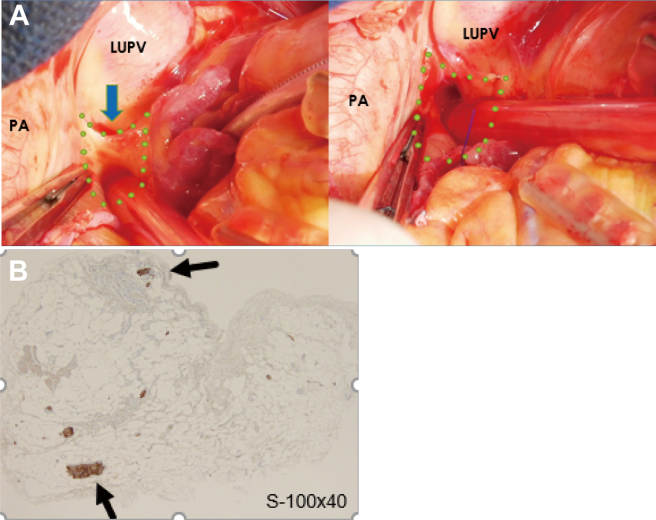
1 Resection of Marshall ligament in ganglionated plexus(GP) A PA: pulmonary artery LUPV: left upper pulmonary vein Blue arrow is Marshall ligament. B The microscopic photo of resected Marshall ligament. Black arrow is the ganglion and sympathetic nerve fibers.

Recently, we have also begun to intervene in the autonomic plexus around the heart to prevent POAF. The ganglionated plexus, an autonomic plexus around the heart, contains a certain percentage of parasympathetic and sympathetic nerves, both of which interact with the atrial muscle. The ganglionated plexus is called the “brain of the heart” because it independently acts on the atrial muscle and is distributed in mainly five locations around the left atrium^41, 42)^. (Figure 9) We have performed resection of the Marshall ligament and the ganglionated plexus located in the upper left atrial wall from the left superior pulmonary vein. The resected tissue showed exchange ganglion and sympathetic nerve fibers, and we are continuing follow-up studies to determine the preventive effect on AF (Figures 10 and 11).

**Figure 12:**
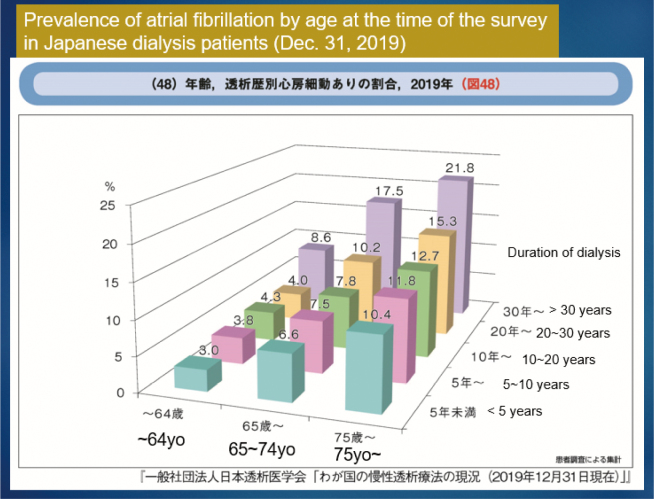
Prevalence of atrial fibrillation by age at the time of the survey in Japanese dialysis patients (Dec. 31, 2019) Reprinted from An Overview of Regular Dialysis Treatment in Japan. 2019 Report, The Japanese Society for Dialysis Therapy 2020: https://docs.jsdt.or.jp/overview/index.html

It was found that renal dysfunction, especially in hemodialysis patients, is frequently complicated by AF^43)^, and more than 11% of hemodialysis patients over 75 years of age have chronic AF (Figure 12)^44)^. Cerebral hemorrhage and stroke are common in dialysis patients because of hypertension caused by renal failure and arteriosclerotic changes in cerebral blood vessels. Furthermore, neurologists, cardiologists, and hemodialysis physicians have different thoughts on anticoagulation medication than hemodialysis patients, making it challenging to continue medical therapy^45, 46)^. Based on this evidence, we believe that aggressive intervention is necessary for LAA in dialysis patients.

**Conclusion and Future Perspectives.** The following is a summary of the treatment methods we have been practicing for patients with ischemic heart disease.

1) Introduction of OPCAB as a minimally invasive procedure: making OPCAB a standard surgical procedure

2) Prevention of long-term cardiac events: the use of multiple arterial grafts

3) A new concept for diabetic patients: Prevention of infection and improvement of prognosis in the long-term follow-up

4) Use in patients with chronic kidney disease: innovations in revascularization, especially for dialysis patients

5) Prevention and management of POAF

6) Freedom from cerebral infarction in the perioperative and lasting lifetime

7) Approaches to POAF

We have continued to devise and improve on these goals for 20 years and have established the Juntendo surgical method today as the standard surgical method in Japan. We will continue to improve long-term outcomes for patients with long-term ischemic heart disease, preventing cardiogenic stroke as our most important target. Furthermore, if advances in imaging allow artificial intelligence to predict long-term outcomes, graft selection and LAA management will be more efficient for all surgeons.

I have upheld two mottos throughout my professional career: “一途一心,” to do what we can do in a lifetime with a passion to last a lifetime, and “一視同仁,” to love every human being with impartiality.

It is my hope that the Department of Cardiovascular Surgery will carry on with such virtues to overcome challenges and have greater accomplishments in the future.

## Funding

No funding was received.

## Author Contributions

AA supervised the work in the Department of Cardiovascular Surgery at Juntendo University.

## Conflicts of Interest Statement

The Author declares that there are no conflicts of interest.
